# High serum magnesium levels are associated with favorable prognoses in diabetic hemodialysis patients, retrospective observational study

**DOI:** 10.1371/journal.pone.0238763

**Published:** 2020-09-17

**Authors:** Chie Ogawa, Ken Tsuchiya, Kunimi Maeda

**Affiliations:** 1 Maeda Institute of Renal Research, Kawasaki, Kanagawa, Japan; 2 Biomarker Society, INC, Kawasaki, Kanagawa, Japan; 3 Department of Blood Purification, Tokyo Women’s Medical University, Tokyo, Japan; International University of Health and Welfare, School of Medicine, JAPAN

## Abstract

**Background:**

Recent studies have found hypomagnesemia is linked to a heightened risk of cardiovascular events and mortality in hemodialysis (HD) patients; however, the level of serum magnesium (s-Mg) necessary for promoting overall health in these patients and the effects of s-Mg in diabetes HD patients remains to be clarified.

**Methods:**

HD outpatients (n = 148 under, age ≤ 70 y) were followed over a 6-y period. They were divided into four groups according to their average s-Mg during the first year (L; low level, H; high level) and if they had DM or not (non-DM). The endpoint was mortality and hospitalization for decline of Activities of Daily Living (death/hospitalization). A receiver operating characteristics curve was used in diagnostic tests to identify s-Mg associated with this endpoint. Kaplan–Meier, log-rank test, and a Cox proportional hazards model were used to evaluate prognoses. Fisher's exact test and multiple regressions examined the causes of the endpoints between the four groups and the factors predictive of s-Mg.

**Results:**

s-Mg at 2.7 mg/dL was associated with death/hospitalization. The 5-y survival rate was 38.1%, 86.7%, 73.2% and 87.5%, in the DM/Mg(L), DM/Mg(H), non-DM/Mg(L) and non-DM/Mg(H) groups, respectively (*P* < 0.001). The Cox proportional hazards model showed significantly lower risk in other groups compared with that in the DM/Mg(L) group [DM/Mg(H); hazard ratio (HR): 0.22, 95% confidence interval (CI): 0.05–0.97, *P* = 0.046, non-DM/Mg(L); HR: 0.32, 95% CI: 0.15–0.68, *P* = 0.003, non-DM/Mg(H); HR: 0.17, 95% CI: 0.06–0.44, *P* < 0.001]. The frequency of the different causes of the endpoints for each group was not significant; s-Mg only associated with age in the DM group.

**Conclusions:**

s-Mg greater than 2.7 mg/dL associated with a favorable prognosis in HD patients with DM, suggesting that s-Mg is a factor independent of diabetes.

## Introduction

Magnesium (Mg) is the fourth most abundant “cation” in the body, serving as a cofactor in over 300 different enzymatic reactions and playing a vital role in a variety of biological processes, including energy production, DNA and RNA synthesis, muscle contraction, vasodilation, neurotransmission and glucose metabolism. Approximately 50%–60% of Mg resides in bones, with the remaining 40% in muscle, brain and nervous tissues. Notably, the vast majority is located in cells, and so serum levels (s-Mg) are hardly reflective of how much Mg is present in the whole body. Indicators of deficiency, nevertheless, can manifest through a variety of symptoms, such as general malaise and loss of appetite, muscle spasms, arrhythmia, hypertension, coronary artery spasms, convulsions, depression, memory deterioration, and disorders of lipid, glucose, and bone metabolism [1, 2].

Ingested Mg is absorbed through the small intestine, excreted through the kidney, and stored in the bone. Typically, 30%–50% of dietary Mg (~120 mg) is absorbed via the intestinal tract and 100 mg is excreted via the kidney. When Mg intake is low, absorption increases to 80%, renal excretion reduces to <0.5% and Mg is pulled from the bone. In HD patients, renal excretion is virtually abolished, and so there has been concern that Mg may be accumulating in them. In recent years, however, hypomagnesemia has become an even greater concern due to the avoidance of medications and supplements with Mg, as well as exposure to low-Mg dialysis solutions. Contemporary studies have reported that hypomagnesemia associates with all-cause mortality [3] and cardiovascular mortality [4] in HD patients, in particular, and others have shown its associations to sudden death [5], bone fractures [6], and arteriosclerosis [7].

Diabetic nephropathy is the most common reason for putting a patient on dialysis, which underlies the initiation of dialysis in 30%–50% of patients in Europe and the United States according to recent DOPPS data and in 39% of patients according to the statistical survey conducted at the end of 2017 by the JSDT. According to a 2015 survey conducted by the Japanese Society for Dialysis Therapy (JSDT), the average length of time on dialysis (dialysis vintage) is 5.03 and 9.40 y in HD patients with and without diabetes mellitus (DM), respectively. Rates of previous cardiovascular disorders have also been observed to be considerably higher in HD patients with DM than those without DM, specifically for myocardial infarctions (14.1% vs. 6.1%), cerebral infarctions (23.0% vs. 13.0%) and amputation of an extremity (6.9% vs. 1.0%). Furthermore, previous studies have shown that control over s-Mg not only improves glycemia and insulin resistance in DM patients, but also reduces their risk for microangiopathy and macroangiopathy [8, 9].

Against this background, we investigated the effect of s-Mg on prognoses in HD patients with DM as the underlying disease.

## Materials and methods

### Patients

This study enrolled a total of 148 maintenance dialysis patients (age ≤ 70 y) who were receiving maintenance HD as outpatients at the Maeda Institute of Renal Research (Kanagawa, Japan) between January 2009 and December 2014. Those who had acute heart disease, cerebrovascular disease, active infection, or malignant tumor at the initiation of the study were excluded. They underwent three 4–5 h long sessions of hemodialysis each week, and all dialysis solutions contained 0.5 mmol/L of Mg.

All patients provided informed consent permitting data sampling and analysis at the time of initiation of the dialysis therapy.

### Methods

Blood was collected before starting the first dialysis session each week. Hematological indices and serum calcium (Ca) and phosphate (P) levels were measured twice a month; levels of serum Mg (s-Mg), serum albumin (s-Alb), and C-reactive protein (CRP) were measured once a month; intact parathyroid hormone levels (PTH) were measured every three months. For DM patients, HbA1c levels were measured once a month. Erythropoiesis-stimulating agents’ preparations and iron replacement therapy were used to control the Hb level with a target level of 10–11 g/dL, according to the guidelines of JSDT. Blood pressure (BP) was measured before each dialysis session. Corrected serum Ca levels (c-Ca) were calculated using Payne's formula [serum Ca + (4.0 − s-Alb)], and normalized protein catabolic rate (nPCR) [10] was calculated once a month. We also surveyed the use of any proton pump inhibitor (PPI) or vitamin D (VitD) receptor antagonist. The endpoint was all-cause mortality or hospitalization due to difficulty in home care or going to hemodialysis for decreased Activities of Daily Living (death/hospitalization).

Patients were divided into four groups, depending on whether they had diabetic nephropathy (DM) or not (non-DM), and whether their s-Mg was at or below (Mg(L)) or above (Mg(H)) the cutoff point for the endpoint [DM/Mg(L) group; n = 21, DM/Mg(H) group; n = 15, non-DM/Mg(L) group; n = 56 and non-DM/Mg(H) group; n = 56]. The statistical power calculation on log-rank test showed 78% by Lakatos’s method [11]. All provided written informed consent.

The “ethics committee of the Biomarker Society, INC' consists of a committee of experts that reviewed and approved your study.

### Statistical analysis

Hematological and biochemical indices, as well as BP values, collected from January to December 2009 were used for the analyses (note: annual mean s-Mg was always used, given evidence of seasonal variations in s-Mg level [12]). To determine s-Mg associated with death/hospitalization, a receiver operating characteristic (ROC) curve with Youden’s index was plotted. One-way analysis of variance compared groups with normally distributed continuous variables, and the Kruskal–Wallis H-test was used for skewed continuous variables. Chi-square tests compared nominally scaled variables. Curves for the cumulative probabilities of time to event were estimated using the Kaplan–Meier product–limit function, and the difference between the curves was determined using the log-rank test. The Cox proportional hazards model was applied to evaluate the impact of DM/non-DM and s-Mg on death/hospitalization. It included the following covariates: age, duration of HD, systolic BP, s-Alb, CRP, and nPCR. Regression diagnostic analyses examined main effects, interactions, and multicollinearity in this model. Causes of death/hospitalization were analyzed by Fisher’s exact test, and multiple regression analyses examined factors related to s-Mg. Data are presented as mean ± standard deviation and as medians with interquartile ranges. Two-tailed *P* < 0.05 was considered statistically significant. All analyses were performed using SAS system software, v. 9.3 (SAS Institute, Cary, NC, USA).

## Results

### Patients

Table 1 shows that of the 148 patients, 101 were male and 47 were female. The mean age was 56.4 ± 10.5 y, and the median duration of dialysis was 9.9 [5.7–16.6] y. Underlying kidney diseases were chronic glomerulonephritis (n = 82, 55.4%), diabetic nephropathy (n = 36, 24.3%), nephrosclerosis (n = 8, 5.4%), and others (n = 22, 14.9%).

**Table 1:** Patient characteristics.

Variables	Total	DM (+)		DM (-)		P-value
		Mg ≤ 2.7	Mg > 2.7	Mg ≤ 2.7	Mg > 2.7	
	n = 148	21	15	56	56	
Mg (mg/dL)	2.7±0.3	2.4±0.2	3.1±0.3	2.5±0.2	3.0±0.2	< .001
	(1.9–3.8)	(1.9–2.7)	(2.8–3.8)	(1.9–2.7)	(2.8–3.6)	
Age (years)	56.4±10.5	64.1±5.8	53.9±8.6	57.5±10.3	52.9±11.0	< .001
Gender (Men)	101(68.2%)	16(76.2%)	10(66.7%)	36(64.3%)	39(69.6%)	0.796
Duration of HD* (year)	9.9[5.7–16.6]	3.6[1.6–7.9]	5.8[2.2–7.9]	10.6[7.9–19.5]	13.4[7.2–17.5]	< .001
Primary diagnosis						
Chronic glomerulonephritis	82(55.4%)	.	.	39(69.6%)	43(76.8%)	
Renal sclerosis	8(5.4%)	.	.	4(7.1%)	4(7.1%)	
Polycystic Kidney	5(3.4%)	.	.	4(7.1%)	1(1.8%)	
RPGN	3(2.0%)	.	.	2(3.6%)	1(1.8%)	
Other	14(9.5%)	.	.	7(12.5%)	7(12.5%)	
systolic BP (mmHg)	149.2±17.0	157.2±10.7	161.6±18.6	143.4±17.9	148.6±14.9	< .001
diastolic BP (mmHg)	82.1±9.7	80.9±6.7	84.1±10.8	81.0±10.4	83.1±9.6	0.530
Hb (g/dL)	10.8±0.7	10.8±0.6	10.8±1.0	10.8±0.6	10.9±0.6	0.951
P (mg/dL)	5.7±0.8	5.3±0.9	5.6±0.6	5.8±0.8	5.7±0.7	0.130
c-Ca (mg/dL)	9.6±0.6	9.5±0.6	9.6±0.5	9.6±0.6	9.6±0.6	0.938
intact-PTH (pg/mL)	213.5±107.5	244.1±101.6	177.2±60.5	216.2±106.7	209±118.7	0.318
s-Alb (g/dL)	3.8±0.3	3.7±0.2	3.9±0.2	3.7±0.3	3.9±0.4	0.020
CRP (mg/d)*	0.1[0.04–0.3]	0.26[0.07–0.70]	0.04[0.02–0.19]	0.17[0.07–0.42]	0.05[0.02–0.16]	< .001
n-PCR (g/kg/day)	0.9±0.1	0.82±0.10	0.95±0.14	0.94±0.13	0.98±0.13	< .001
PPI	63(42.6%)	9(42.9%)	4(26.7%)	33(58.9%)	17(30.4%)	0.012
Vit D	110(74.3%)	14(66.7%)	9(60.0%)	42(75.0%)	45(80.4%)	0.329
HgA1c (%)	.	5.6±0.7	6.0±0.7	.	.	0.121
Endpoint	37(25.0%)	13(61.9%)	2(13.3%)	15(26.8%)	7(12.5%)	< .001

Mean ± SD and median and interquartile range [IQR]*.

Abbreviations: DM, diabetes; HD, hemodialysis, RPGN, rapidly progressive glomerulonephritis; BP, blood pressure; Hb, hemoglobin; c-Ca; corrected Ca; PTH; parathyroid hormone; s-Alb; serum albumin; CRP; c reactive protein; n-PCR; normalized protein catabolic rate; PPI, proton pump inhibitor; Vit D, vitamin D receptor antagonist.

Age was higher in the DM/Mg(L) group than the non-DM/Mg(H) group (*P* < 0.001). Duration of HD was longer in the non-DM/Mg(H) group than the DM/Mg(L) group (*P* < 0.001). Systolic BP was higher in the DM groups than the non-DM groups (*P* < 0.001). s-Alb was higher in the Mg(H) groups than the Mg(L) groups (*P* = 0.020). CRP was higher in the DM/Mg(L) group than the DM/Mg(H) group (*P* < 0.001). nPCR was higher in the non-DM/Mg(H) group than the DM/Mg(L) group (*P* < 0.001). The rate of PPI use was higher in Mg(L) groups than the Mg(H) groups.

### Distribution of s-Mg

**Fig 1:**
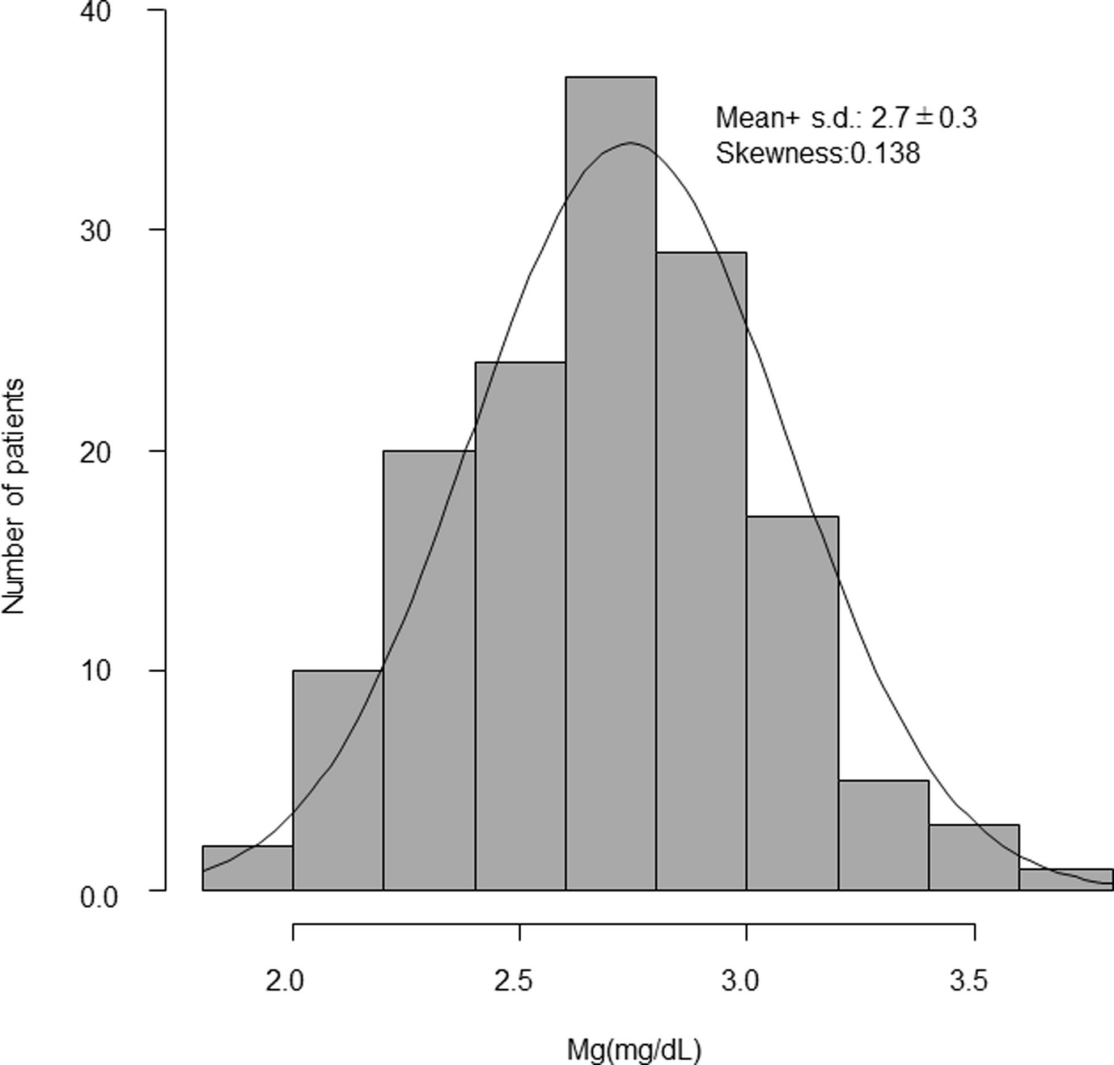
Distribution of mean serum Mg value during the first year.

Fig 1 shows that s-Mg had a nearly normal distribution with a mean of 2.7 mg/dL (skewness: 0.14, kurtosis: 0.13). The mean range for the first year was 1.9–3.8 mg/dL.

### ROC analysis

**Fig 2:**
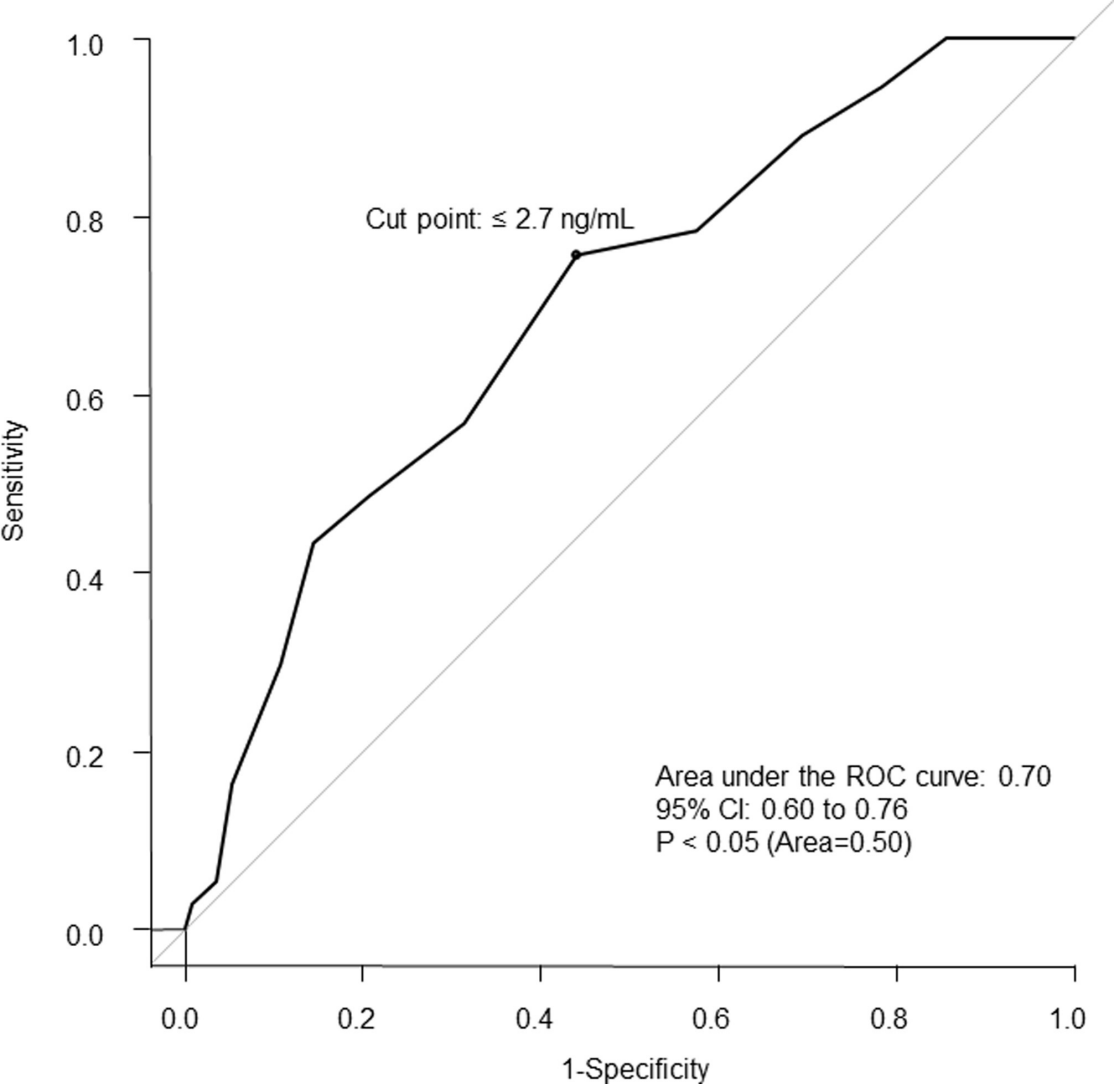
The ROC curve of serum Mg with all-cause death or hospitalization for decline of ADL.

Fig 2 shows that the cutoff point for s-Mg was less than 2.7 mg/dL (sensitivity: 75.7%, specificity: 55.9%, area under the curve: 0.70, 95% confidence interval [CI]: 0.60–0.76, *P* < 0.05).

### Investigation of the 5-y survival rate

**Fig 3:**
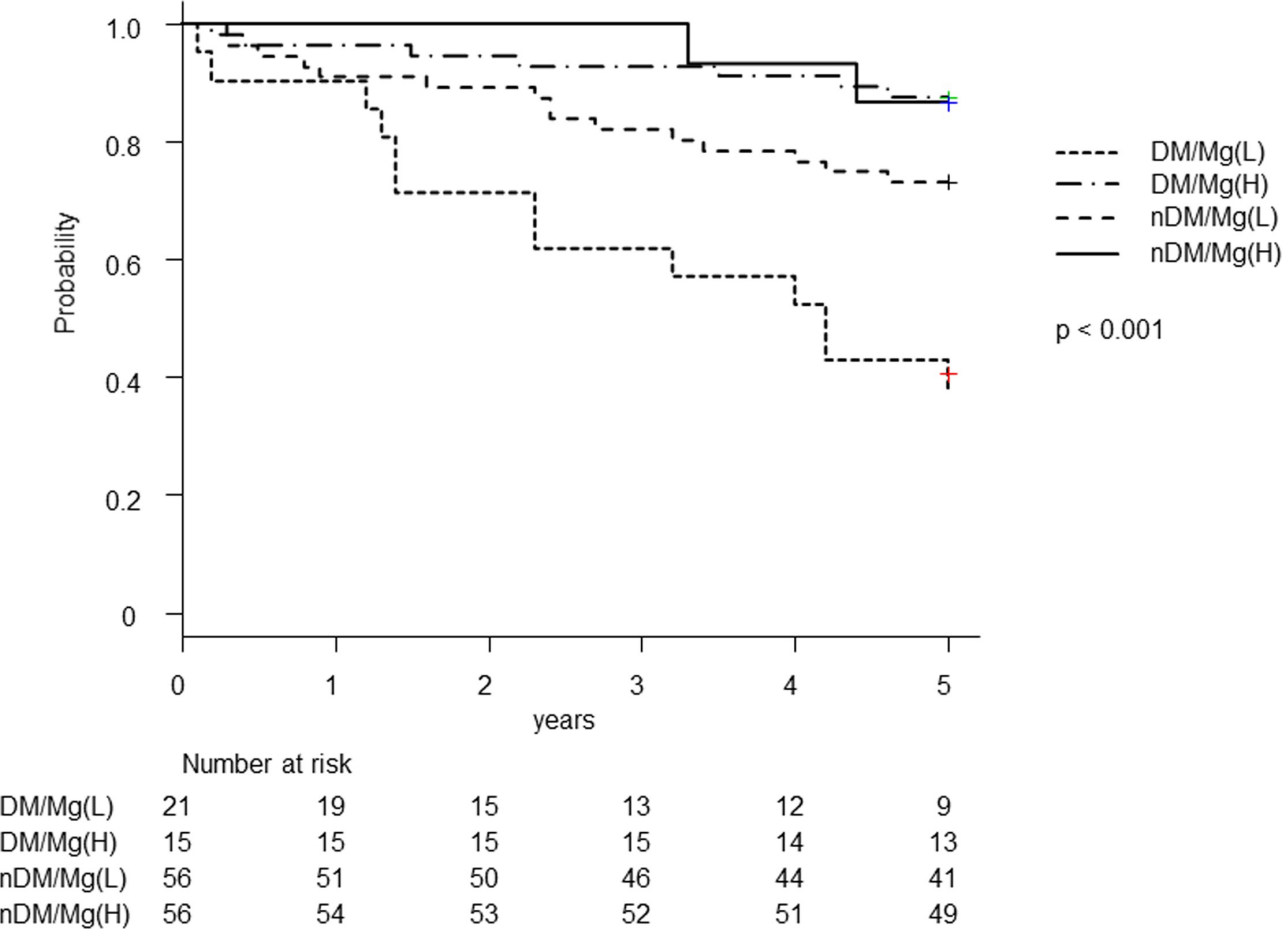
Kaplan–Meier product–limit function estimates for time-to-event analysis by the four groups. The endpoint was all-cause death or hospitalization for decline of ADL. The 5-y survival rate in the DM/Mg(L) group was noted to be significantly low (*P* < 0.001).

**Fig 4:**
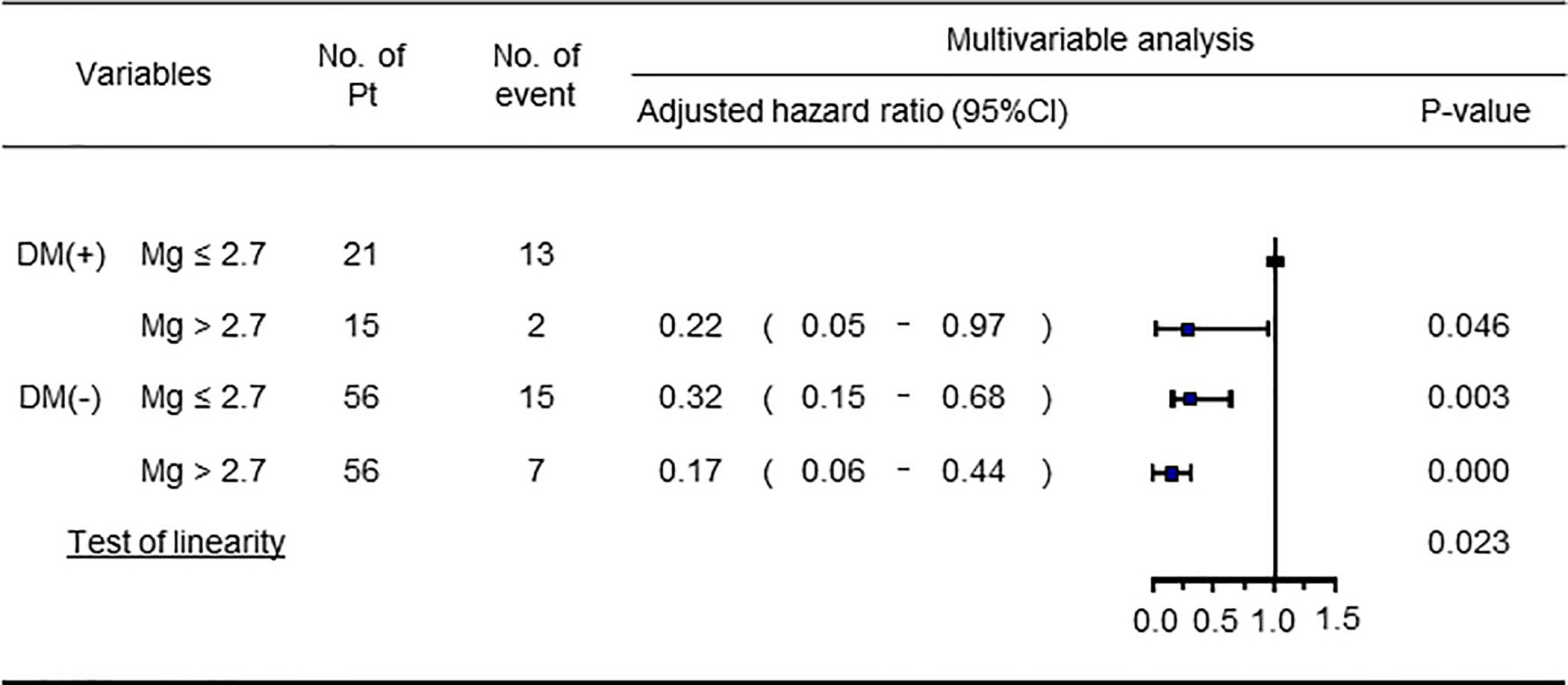
The multivariable Cox proportional hazards model. Adjusted hazard ratios were obtained by the multivariable model using the imbalanced covariates: age, duration of hemodialysis, systolic blood pressure, serum albumin levels, C-reactive protein, and normalized protein catabolic rate.

Among the 148 patients, 21 (14.2%) died and 16 (10.8%) were hospitalized for decline of Activities of Daily Living. Fig 3 shows that the 5-y survival rate was highest for the non-DM/Mg(H) group (87.5%), followed by the DM/Mg(H) group (86.7%), the non-DM/Mg(L) group (73.2%), and finally the DM/Mg(L) group (38.1%) (*P* < 0.001). Fig 4 shows the Cox proportional hazards model revealed significantly lower hazard ratios (HRs) for the DM/Mg(H) group [HRs 0.22 (95% confidence interval (CI) 0.05–0.97), *P* = 0.046], the non-DM/Mg(L) group [0.32 (95% CI: 0.15–0.68), *P* = 0.003], and the non-DM/Mg(H) group [0.17 (95% CI: 0.06–0.44), *P* < 0.001].

### Investigation of death/hospitalization

Table 2 shows that the causes of death/hospitalization included cardiovascular disease in 10 patients (6.8%), infectious disease in 3 (2.0%), malignancy in 4 (2.7%), and sudden death in 4 (2.7%). The DM/Mg(L) group experienced the highest number of death/hospitalization cases (n = 13), with 5 (23.8%) dying of cardiovascular disease, 1 (4.8%) of malignancy, and 2 (9.5%) of sudden death. In contrast, the non-DM/Mg(H) group had the lowest number of death/hospitalization cases, with 2 dying of infection and 1 of malignancy.

**Table 2:** Investigation of the causes of death or hospitalization for decline of ADL.

	Total	DM (+)		DM (-)		P-value
		Mg ≤ 2.7	Mg >2.7	Mg ≤ 2.7	Mg > 2.7	
	(N = 148)	(N = 21)	(N = 15)	(N = 56)	(N = 56)	
All deceased patients	37(25.0%)	13(61.9%)	2(13.3%)	15(26.8%)	7(12.5%)	
Cause of death/ hospitalization						
Cardiovascular disease	10(6.8%)	5(23.8%)	2(13.3%)	3(5.4%)	.	0.538
Infectious disease	3(2.0%)	.	.	1(1.8%)	2(3.6%)	
Malignancy	4(2.7%)	1(4.8%)	.	2(3.6%)	1(1.8%)	
Sudden death	4(2.7%)	2(9.5%)	.	2(3.6%)	.	
Other	11(8.0%)	4(19.0%)	.	4(7.1%)	3(5.4%)	
Unknown	5(3.4%)	1(4.8%)		3(5.4%)	1(1.8%)	

P-value for the comparison among four groups.

### Factors related to s-Mg

Table 3 shows there was a negative correlation between age and s-Mg, with β-coefficients of −0.008 (95% CI: −0.014 to −0.002, *P* < 0.001), and a positive correlation between s-Mg and both s-Alb and c-Ca, with β-coefficients of 0.267 (95% CI: 0.069 to 0.464, *P* < 0.001) and 0.100 (95% CI: 0.003 to 0.195, *P* = 0.044), respectively. Only age was associated with s-Mg in DM patients [β-coefficient of −0.022 (95% CI; −0.038 to −0.005, *P* = 0.009)], and only s-Alb was associated with s-Mg in non-DM patients (β-coefficients of 0.305, 95% CI; 0.058 to 0.471, *P* = 0.013).

**Table 3:** The relationship for factors with s-Mg level by multiple linear regression model.

	Independent variables	No. of patients	Adjusted regression β-coefficient		95% confidence interval				P-value
All	age	148	-0.008	(	-0.014	to	-0.002	)	< .001
	s-Alb		0.267	(	0.069	to	0.464	)	< .001
	c-Ca		0.100	(	0.003	to	0.195	)	0.044
DM	age	36	-0.022	(	-0.038	to	-0.005	)	0.009
	s-Alb		0.328	(	-0.238	to	0.893	)	0.246
	c-Ca		0.114	(	-0.107	to	0.336	)	0.301
non-DM	age	112	-0.005	(	-0.011	to	0.002	)	0.137
	s-Alb		0.305	(	0.058	to	0.471	)	0.013
	c-Ca		0.081	(	-0.025	to	0.187	)	0.132

All; all patients, DM; patients with diabetes, non-DM; patients with no diabetes, Age (per 1year increase), s-Alb (per 1g/dL increase), c-Ca (per 1mg/dL increase).

## Discussion

Within the body, Mg is almost exclusively found intracellularly. Mg in serum accounts for only a very small portion of total Mg, and this is measured in routine laboratory tests. The range of s-Mg in healthy individuals is generally 0.75–1.0 mmol/L (1.8–2.4 mg/dL) [13], though some reports recommend using 0.82–0.85 mmol/L (2.0 mg/dL) as the lower limit in DM patients indicative of normal renal function [14, 15]. For HD patients, previous studies have reported an increased risk of death by cardiovascular disease and overall mortality when s-Mg is ≤2.5–3.1 mg/dL [3–5, 16–18]. In this study, we examined s-Mg in HD patients, and found a normal distribution with a range of 1.9–3.8 mg/dL and a mean of 2.7 mg/d, similar to findings from >140,000 JSDT data reporting a mean of 2.6 mg/dL) [4]. In HD patients, acidosis is corrected by HD; pH has been known to affect the ability of Mg to bind to albumin [19], and ionized Mg and anion-bound Mg are dialyzable. Moreover, as HD patients have disordered mineral and bone metabolism, they not only have deteriorated excretory function but also are in a state of Mg metabolism different from that in healthy individuals. These conditions may underlie the optimal s-Mg level for HD patients.

Participants in the non-DM/Mg(H) group were found to have a significantly better prognosis than the DM/Mg(L) group. The multivariate analysis produced similar results when adjustment was made for age, systolic hypertension, s-Alb, CRP, dialysis vintage, and nPCR, which significantly differed between groups, or for sex, diastolic hypertension, Hb, corrected serum Ca, P, and intact PTH, which were suggested to be associated with prognosis in HD patients. So far, hypomagnesemia has been reported to be associated with poor prognosis in HD patients and risk for microangiopathy and macroangiopathy in DM patients, respectively. However, none have examined the effects of Mg on the prognosis of HD patient with DM and been compared with that of HD patient with non-DM. Interestingly, the results revealed that the survival rate in the DM/Mg(H) group was higher than that in the non-DM/Mg(L) group, suggesting that prognosis may be improved by maintaining s-Mg at a higher level, even in patients with DM.

Our analysis suggested that death/hospitalization was elevated in patients with s-Mg of ≤2.7 mg/dL. With regard to the causes of this endpoint, cardiovascular disorders were found to be the most common (23.8%) cause of death/hospitalization in the DM/Mg(L) group, nearly twice as high as that observed in the DM/Mg(H) group. These disorders accounted for 5.4% of death/hospitalization in the non-DM/Mg(L) group, but were non-existent in the non-DM/Mg(H) group. In other words, cardiovascular disorders were found more frequently in the DM groups compared with the non-DM groups, and were less frequent in the Mg(H) subgroup compared with the Mg(L) subgroup. The result of the present analysis also suggests that hypomagnesemia is associated with cardiovascular disorders, although there was no significant difference between the groups as a cause of endpoint.

Arteriosclerosis in HD patients is characterized by the thickening and calcification of the arterial walls, though *in vivo* studies show Mg is capable of inhibiting this process [20]. For those who suffer from hypomagnesemia, a Mg formulation was found to improve thickness of the carotid intima-media [21]. In addition, while hyperphosphatemia is widely known to be strongly involved in arterial calcification in HD patients, mortality from cardiovascular disorders has been reported to decrease when s-Mg is high, even if the P level is high [22]. Collectively, these reports show case the importance of s-Mg control for cardiovascular risk management in HD patients. In our study, sudden death was observed only in the low Mg group, and it is possible this was due to hypomagnesemia. Further investigations are warranted since the case number was small.

For all patients, age and s-Alb were found to be negatively correlated and positively correlated, respectively, to s-Mg. Given that Mg comes from the food we eat, this suggests that decreases in s-Mg may be a result of reductions in food intake and compromised intestinal absorption. As a result of recent changes in dietary patterns, Mg intake has markedly declined even in healthy people, and intakes in Europe and the United States are lower than the recommended amounts [23–26]. In Japan, the 2019 National Health and Nutrition Survey revealed that the Mg intake was well below the guidelines for people in their 50s and 60s. Many foods abundant in Mg, such as whole-grain cereals, legumes, nuts, and chocolate, are restricted for HD patients because they possess phosphorus, potassium and protein, which are deleterious to their health. As a result, dietary Mg intakes, and therefore s-Mg, tend to be especially poor in these individuals. For patients with renal disease, a common comorbidity to DM, one study showed they have reduced intestinal absorption of Mg [27]. In our study, age was the only factor showing an association with s-Mg for the DM group, and s-Alb was the only factor showing an association with s-Mg for the non-DM group. These suggest that s-Mg does not decrease even in elderly individuals, as long as their nutritional status is good. Among HD patients, the prevalence of DM in elderly patients is increasing, so studies on appropriate Mg supplementation are warranted to improve the prognosis.

Nodding to supplementary analyses, s-Mg was positively correlated with c-Ca. This is not surprising, since Ca metabolism is closely related to Mg metabolism, and intestinal absorption of Ca and Mg is facilitated by VitD and PTH. Mg deficiency reduces PTH expression due to hypocalcemia, and therefore impacts bone and Ca metabolism [2]. Indeed, hypocalcemia tends to coincide with hypomagnesemia, although no association was detected between s-Mg and VitD or intact PTH in this study. Regarding PPIs, previous investigations show they reduce Mg absorption in HD patients [28, 29], and in our study, a significantly larger portion of patients in the Mg(L) group were oral PPI users.

### Limitations

This study had several limitations. Because this was a retrospective and observational study and the number of enrolled patients was small, there is the possibility that there may be other confounding factors that remain to be analyzed.

## Conclusion

We discovered that the optimal s-Mg in HD patients was higher than that in healthy individuals and that s-Mg was a prognostic determinant in HD patients with DM as the underlying disease. We also found that the survival rate in HD patients with DM was similar to that in patients without DM when s-Mg was maintained at a level higher than that in healthy individuals. On the whole, these findings demonstrate that successful control of Mg levels contributes greatly to better prognoses for HD patients with DM. Future investigations on Mg supplementation are necessary since hypomagnesemia was linked to age-related malnutrition and oral PPI use, and large-scale studies will be helpful in confirming the present results.
